# A Spike-destructing human antibody effectively neutralizes Omicron-included SARS-CoV-2 variants with therapeutic efficacy

**DOI:** 10.1371/journal.ppat.1011085

**Published:** 2023-01-27

**Authors:** Lu Meng, Jialu Zha, Bingjie Zhou, Long Cao, Congli Jiang, Yuanfei Zhu, Teng Li, Lu Lu, Junqi Zhang, Heng Yang, Jian Feng, Zhifeng Gu, Hong Tang, Lubin Jiang, Dianfan Li, Dimitri Lavillette, Xiaoming Zhang

**Affiliations:** 1 Key Laboratory of Molecular Virology & Immunology, Institut Pasteur of Shanghai, Chinese Academy of Sciences/University of Chinese Academy of Sciences, Shanghai, China; 2 CAS Center for Excellence in Molecular Cell Science, Shanghai Institute of Biochemistry and Cell Biology, Chinese Academy of Sciences (CAS), Shanghai, China; 3 Shenzhen Kangtai Biological Products Co., Shenzhen, China; 4 Key Laboratory of Medical Molecular Virology (MOE/NHC/CAMS), Department of Medical Microbiology and Parasitology, School of Basic Medical Sciences, Shanghai Institute of Infectious Diseases ad Biosecurity, Shanghai Medical College, Fudan University, Shanghai, China; 5 Suzhou Institute of Systems Medicine, Suzhou, China; 6 Affiliated Hospital of Nantong University, Nantong, China; 7 Nanjing Advanced Academy of Life and Health, Nanjing, China; 8 Shanghai Huashan Institute of Microbes and Infections, Shanghai, China; The Ohio State University, UNITED STATES

## Abstract

Neutralizing antibodies (nAbs) are important assets to fight COVID-19, but most existing nAbs lose the activities against Omicron subvariants. Here, we report a human monoclonal antibody (Ab08) isolated from a convalescent patient infected with the prototype strain (Wuhan-Hu-1). Ab08 binds to the receptor-binding domain (RBD) with pico-molar affinity (230 *pM*), effectively neutralizes SARS-CoV-2 and variants of concern (VOCs) including Alpha, Beta, Gamma, Mu, Omicron BA.1 and BA.2, and to a lesser extent for Delta and Omicron BA.4/BA.5 which bear the L452R mutation. Of medical importance, Ab08 shows therapeutic efficacy in SARS-CoV-2-infected hACE2 mice. X-ray crystallography of the Ab08-RBD complex reveals an antibody footprint largely in the β-strand core and away from the ACE2-binding motif. Negative staining electron-microscopy suggests a neutralizing mechanism through which Ab08 destructs the Spike trimer. Together, our work identifies a nAb with therapeutic potential for COVID-19.

## Author summary

To fight against SARS-CoV-2 Omicron subvariants, we screened and selected one human monoclonal antibody (Ab08) previously isolated from a convalescent patient infected with the SARS-CoV-2 prototype strain (Wuhan-Hu-1). The features of Ab08 are as followed: (1) Ab08 can effectively neutralize SARS-CoV-2 and variants of concern (VOCs) including Alpha, Beta, Gamma, Mu, Omicron BA.1 and BA.2, and to a lesser extent, Delta and Omicron BA.4/BA.5. (2) Ab08 can act as a therapeutic agent in SARS-CoV-2-infected hACE2 mice which are highly susceptible to infection. (3) Ab08 could destruct the Spike trimer which is essential for SARS-CoV-2 entry into the host cells. Together, our work identifies an effective neutralizing antibody with therapeutic potential for COVID-19.

## Introduction

The global Coronavirus Disease 2019 (COVID-19) pandemic has caused 652 million infections and 6.65 million deaths according to official statistics (https://www.worldometers.info/coronavirus/2022-12-09). SARS-CoV-2 infection is initiated upon the attachment of the viral transmembrane Spike (S) glycoprotein to the angiotensin-converting enzyme 2 (ACE2) in host cells. The S glycoprotein is a homotrimer with the S1 and S2 subunits in each protomer. The S1 subunit contains an N-terminal domain (NTD) and a receptor-binding domain (RBD) which includes a β-strand core and a receptor-binding motif (RBM) that is largely consisted of loops [1,2]. RBD can adopt either ‘down’ or ‘up’ conformations, with the latter being competent in engaging with ACE2 [3–5]. Till now, four major classes of RBD-targeting monoclonal antibodies (mAbs) have been defined: The Class 1 mAbs target RBM in up-RBD; the Class 2 mAbs bind to RBM in both up- and down-RBDs; the Class 3 mAbs recognize non-RBM epitopes in both up- and down-RBDs; and the Class 4 mAbs bind to non-RBM epitopes in up-RBD [5,6]. In addition, RBD-targeting neutralizing antibodies (nAbs) that are incompatible with either conformations have been reported. Such antibodies including CR3022 [7], EY6A [8], FD20 [9] and 35B5 [10] neutralize SARS-CoV-2 by destroying the S trimer and have little effect on ACE2-binding. By targeting the more conserved β-strand core, these nAbs are generally less sensitive to escape mutants [7–10].

A major reason for the ongoing rage of COVID-19 is the emergence of new variants that are more infectious and capable of breakthrough infection. In particular, the Omicron subvariants have become dominant in recent months. The BA.1 (B.1.1.529.1) subvariant was firstly identified in South Africa in November 2021 [11]. The BA.2 (B.1.1.529.2) subvariant currently dominates the global pandemic, while BA.2.12.1 and BA.4/BA.5 have surged in the United States and South Africa, respectively (https://ourworldindata.org/explorers/coronavirus-data-explorer). The S protein of Omicron BA.1 contains an alarming number of more than 35 mutations, including at least 15 in the RBD [12]. Omicron BA.2 shares 21 mutations with BA.1 in the S protein. In addition, BA.2 contains 8 unique mutations while BA.1 contains 13 unique mutations. Another Omicron subvariant named as BA.1.1 has one more mutation R346K compared to BA.1. Such extensive mutations on RBD are the major mechanism for the breakthrough infection. The serum neutralizing activities of individuals who had received three homologous mRNA vaccinations exhibit mean decreases of 6.5-fold for Omicron BA.1 pseudovirus and >4.1-fold for BA.1 authentic virus compared to the D614G controls [12]. Similar results were observed for the sera or plasma from convalescent patients or vaccinees to several Omicron subvariants BA.1, BA.1.1 and BA.2 [11–16], explaining the emerging clinical data for higher rates of reinfection and vaccine breakthroughs by Omicron subvariants [17,18].

In line with severely compromised neutralizing efficacy of the sera from convalescent patients and vaccinees, the majority of nAbs that have been authorized or approved for clinical use fail to neutralize Omicron subvariants. For instance, REGN10987 (Imdevimab) /REGN10933 (Casirivimab) [19], COV2-2196 (Tixagevimab) [20], LY-CoV555 (Bamlanivimab) [21], CB6 (Etesevimab) [22] and Brii-196 (Amubarvimab) are ineffective against Omicron BA.1, BA.1.1 and BA.2. Although Brii-198 (Romlusevimab) [23] remains active against BA.1, it fails to neutralize BA1.1 and BA2 [12,15]. Similarly, S309 (Sotrovimab) [24] neutralizes BA.1 and BA1.1, but not BA2 [15]. By contrast, COV2-2130 (Cilgavimab) [20] remains effective against BA.2 but not BA.1 or BA1.1. LY-CoV1404 (Bebtelovimab) [25] has thus far been effective against BA.1, BA1.1, BA.2, BA.2.12.1 and BA.4/BA.5 [12,15], but such antibodies are rare.

Here, we report a human neutralizing monoclonal antibody (dubbed Ab08) isolated from a convalescent patient infected with the prototype SARS-CoV-2 strain. Ab08 neutralizes SARS-CoV-2 and Omicron-included variants by destructing the S trimer. Of medical relevance, Ab08 exhibits therapeutic effect in SARS-CoV-2-infected hACE2-transgenic mice. These features make Ab08 a potential therapeutic candidate to fight COVID-19.

## Results

### Ab08 binds RBD with pico-molar affinity and effectively neutralizes the majority of SARS-CoV-2 variants

**Fig 1:**
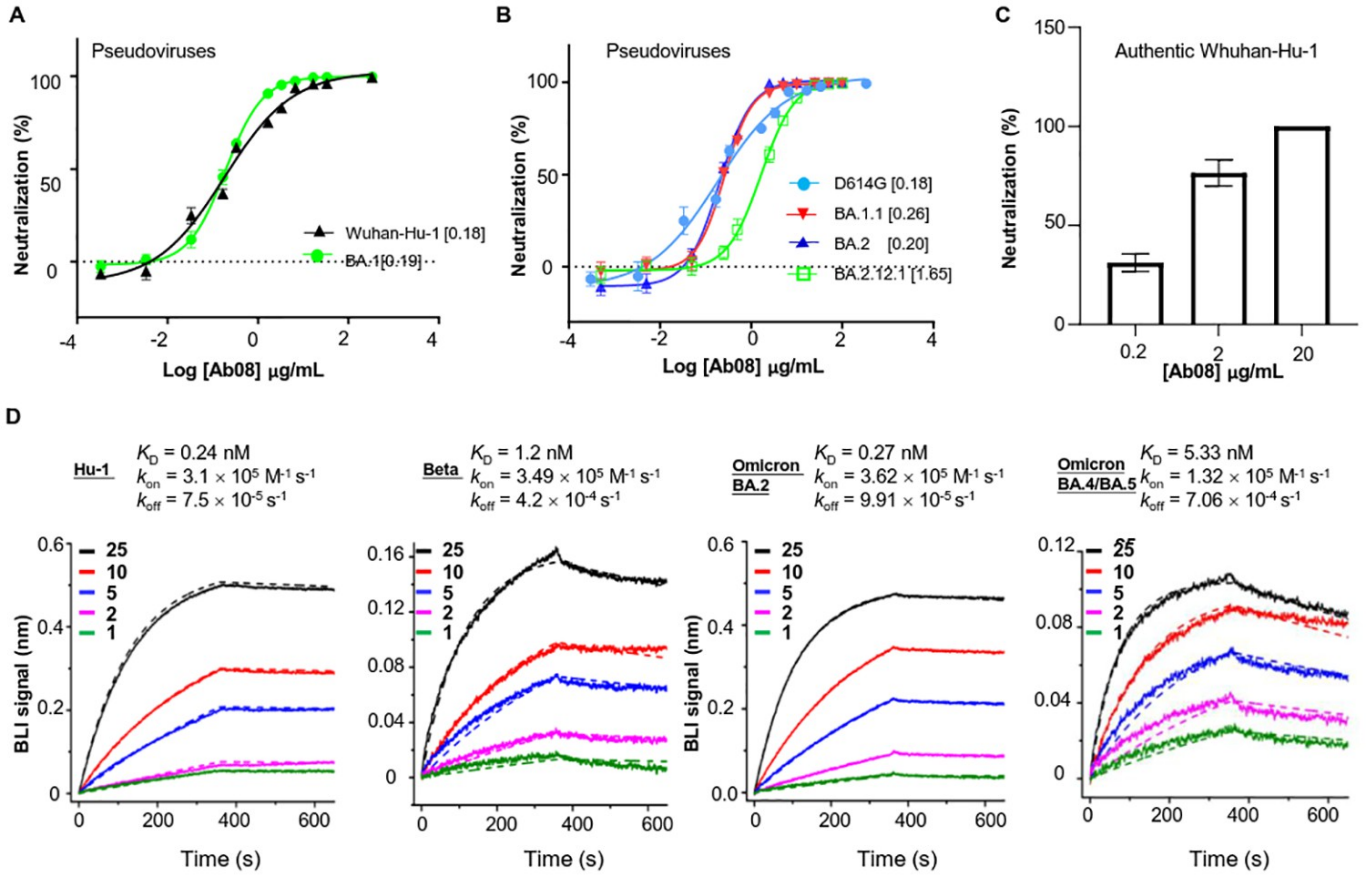
Ab08 neutralizes SARS-CoV-2 Omicron-included variants and binds to RBD with pico-molar affinities. (**A-B**) Neutralization assay of Ab08 using pseudoviruses harboring the S proteins from SARS-CoV-2 Wuhan-Hu-1 and Omicron BA.1 (A) or D614G and Omicron subvariants as indicated (B). Data are plotted as mean ± SEM (SEM: standard error of mean; n = 3 biological replicates). Numbers in brackets indicate IC_50_ values in μg/mL. (**C**) Neutralization activity of Ab08 against authentic SARS-CoV-2. A Plaque Reduction Neutralization Test (PRNT) was applied. Data are plotted as mean ± SEM (n = 3, biological replicates). (**D**) Binding affinities of Ab08 to SARS-CoV-2 prototype and variant RBDs using bilayer interferometry (BLI). Biotinylated RBDs were immobilized on a Streptavidin sensor and Ab08 (scFv) was used as analytes at indicated concentrations (nM). Solid lines represent experimental data and dashed lines represent fitted curve.

Previously, we have isolated a panel of SARS-CoV-2 RBD-binding mAbs from the peripheral B cells of a convalescent patient infected with the prototype strain (Wuhan-Hu-1) during the early phase of the epidemic in 2020. Of these cloned antibodies, Ab08 exhibited potent neutralizing activity against Wuhan-Hu-1 pseudovirus infection (S1 Fig). The destructive spread of the Omicron subvariants prompted us to screen Omicron-neutralizing antibodies from known SARS-CoV-2 neutralizing antibodies. We identified that the Ab08 also showed potent neutralizing capacity against the Omicron BA.1 pseudovirus with the half maximum inhibitory concentration (IC_50_) of 0.19 μg/mL, similar to its activity against Wuhan-Hu-1 pseudovirus with a IC_50_ of 0.18μg/mL (Fig 1A). Ab08 also demonstrated relatively broad activities on the pseudoviruses carrying the D614G mutation (IC_50_ = 0.18 μg/mL), and mutations from Omicron BA.1.1 (IC_50_ = 0.26 μg/mL), BA.2 (IC_50_ = 0.20 μg/mL) and BA.2.12.1 (IC_50_ = 1.65 μg/mL) (Fig 1B). By contrast, the CB6 antibody [22] failed to neutralize the Omicron BA.1 pseudovirus (S2 Fig). We further validated the neutralizing effect of Ab08 on authentic viruses. As shown in Fig 1C, Ab08 could effectively neutralize authentic SARS-CoV-2 with an approximate the highest antibody dilution reducing plaque numbers by 50% (PRNT_50_) at 1.18 μg/mL.

To investigate if Ab08 can neutralize other variants of concern/interest, we constructed MLV pseudotypes harboring the S proteins from B.1.1.7 (Alpha), B.1.351 (Beta), B.1.617.2 (Delta), P.1 (Gamma), and B.1.621 (Mu), and BA.4/BA.5 (Omicron). As shown in S3 Fig, Ab08 displayed similar activities against the majority of the variants, reporting IC_50_ values from 0.18 to 0.45 μg/mL, with the exception of Delta (IC_50_ = 27.70 μg/mL) and Omicron BA.4/BA.5 (IC_50_ >25 μg/mL). The Delta and Omicron BA.4/BA.5 variants share the RBD L452R mutation while the Omicron BA.2.12.1 harbors the RBD L452Q mutation, which suggests that the Leu452 site is key to Ab08 recognition. To directly test this idea, we have constructed several pseudoviruses with various point mutations within SARS-CoV-2 D614G S, including L452R, L452Q and T478K. We identified that the neutralization capacity of Ab08 was mostly affected by the L452R mutation, followed by the L452Q mutation (S4 Fig), thus confirming that Ab08 is susceptible to the Leu452 mutation.

To further characterize Ab08, the binding affinities of Ab08 to prototype and variant RBDs were measured by bio-layer interferometry (BLI). To avoid avidity and bridging effects by the IgG format, the single-chain fragment variable (scFv) of Ab08 was used. The results revealed high binding affinities between Ab08 and prototype and Omicron BA.2 RBDs with the *K*_D_ of 0.24 nM and 0.27 nM, respectively. Of note, the *K*_D_ between Ab08 and Beta, and Omicron BA.4/BA.5 RBDs were 1.2 nM and 5.33 nM, respectively, suggesting a compromised binding affinity of Ab08 to certain variants, particularly the Omicron BA.4/BA.5 (Fig 1D). Ab08 utilized an IGHV1-69*01 and IGLV1-40*01 family with heavy chain-complementarity-determining region (H-CDR)-3, light chain-complementarity-determining region (L-CDR)-3 lengths of 12 and 5 amino-acid residues, respectively. Taken together, we have isolated a potent human neutralizing antibody against Omicron-included SARS-CoV-2 variants with pico-molar affinity to RBD.

### Ab08 exhibits therapeutic efficacy in SARS-CoV-2-infected hACE2 mice

**Fig 2:**
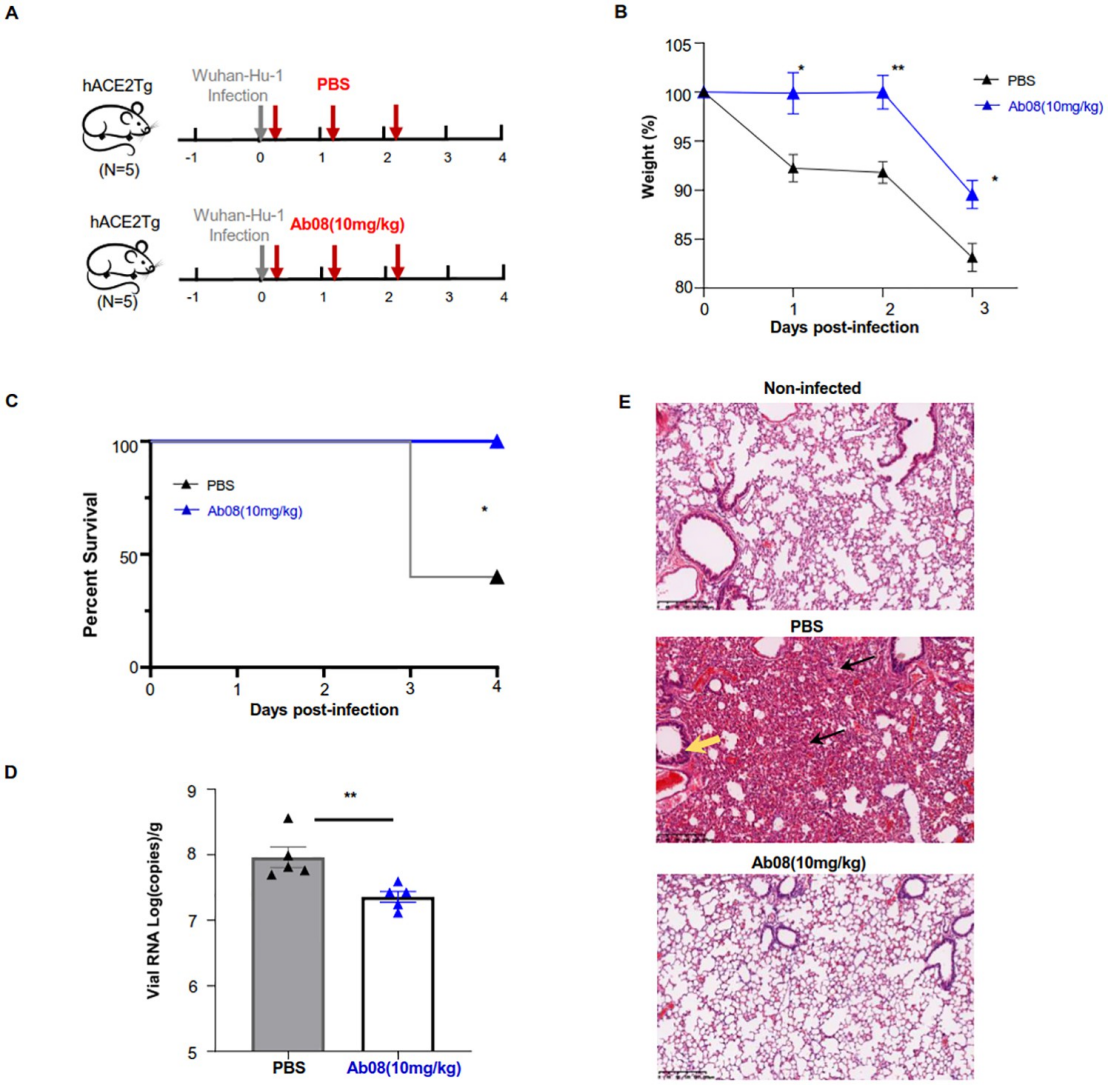
Ab08 shows therapeutic efficacy in hACE2 mice infected with live SARS-CoV-2. (**A**) hACE2 mice intranasally infected with 10^5^ PFU of SARS-CoV-2 were divided into two groups: the Ab08 group was intraperitoneally administered with 10 mg/kg Ab08 (n = 5) and the PBS group was injected with PBS (n = 5) at 4-hour, 28-hour and 52-hour post infection (red arrow). All mice were sacrificed on day 4 post infection. The mouse images are from Openclipart. (**B**) Weight change of mice was monitored daily (n = 5; Unpaired Student’s *t* test: *p<0.05; **p<0.01; symbols denote mean ± SEM.) (**C**) Percent survival was determined for the Ab08 and PBS groups. (n = 5; Kaplan-Meier survival analysis with Log-rank test: * p<0.05). (**D**) SARS-CoV-2 viral RNA loading in lungs of the Ab08 and PBS groups on day 4 post infection. Viral RNA copies were analyzed by real-time qPCR. (Unpaired Student’s *t* test: * p<0.05). (**E**) Representative H&E staining showing pathological changes in the mouse lungs from different groups (Top: Non-infected; Middle: PBS; Bottom: Ab08). The PBS group exhibited severe pneumonia with blocked terminal bronchioles, fibroplasia, and organization (black arrow), and peribronchial and perivascular infiltration (yellow arrow). Scale bars indicate 200 μm.

Neutralizing antibodies are important therapeutics against viral disease, especially for populations unsuitable for vaccination. Currently, anti-SARS-CoV-2 nAbs that target the S protein have shown clinical benefits in treating SARS-CoV-2 infections [26,27]. To assess the therapeutic activity of Ab08 *in vivo*, groups of 8-week-old human ACE2 transgenic (hACE2) mice were intranasally infected with 10^5^ plaque-forming units (PFU) of the authentic SARS-CoV-2 Wuhan-Hu-1 virus. At 4-hour, 28-hour and 52-hour after infection, mice were intraperitoneally injected by either Ab08 (10 mg/kg) or PBS as shown in Fig 2A. All mice were monitored daily for body-weight changes and were sacrificed on day 4 post-infection.

Compared to the Ab08 group, mice from the PBS group showed significant weight loss at day 1, day 2 and day 3 post-infection (Fig 2B). Strikingly, all mice from the Ab08 group survived during the experimental period while 60% (three out of five) of the mice from the PBS group succumbed at day 3 post-infection (Fig 2C). In line with this, the Ab08 group displayed lower viral RNA load compared with the PBS group (Fig 2D). In addition, histopathological examination of the lungs revealed minor signs of pulmonary inflammation and pathological damage in the Ab08 group. By contrast, the PBS group displayed severe inflammatory cell infiltration, alveolar septal thickening, distinctive vascular injury, and focal hemorrhage (Fig 2E). Collectively, these results demonstrate therapeutic efficacy of Ab08 in SARS-CoV-2-infected hACE2 mice.

### Structural analysis reveals Ab08 engaging RBD at an epitope away from RBM

**Fig 3:**
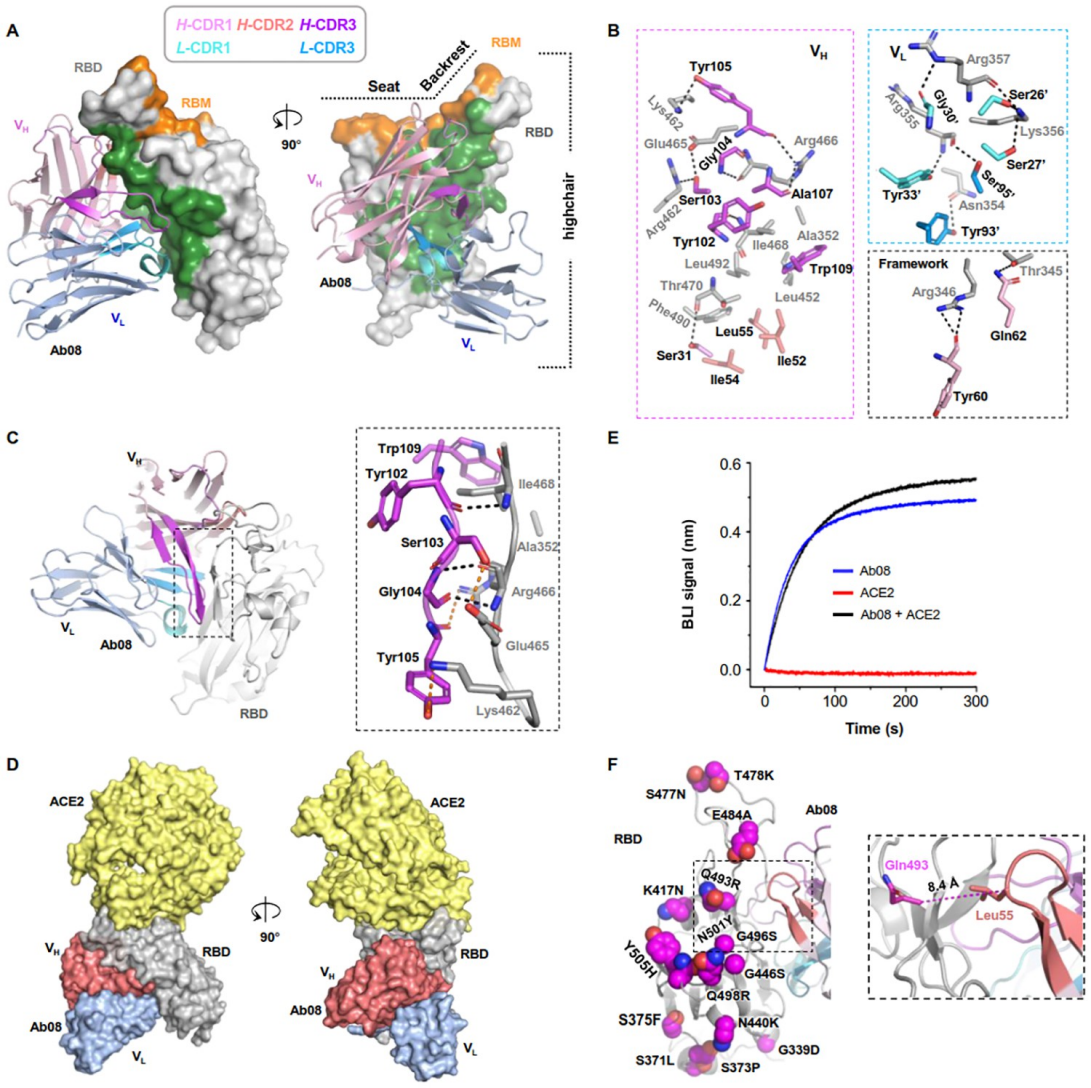
Structural characterization informs mechanism for the relatively broad activity of Ab08. (**A**) The overall structure of the Ab08 (scFv) (cartoon) in complex with the high-chair-shaped RBD (grey surface). The Ab08 epitope is colored green. RBD-interacting CDRs are color-coded as indicated. (**B**) Interactions between RBD (grey) and Ab08. CDR residues are color-coded as in **A**. Ab08 residues are labeled black and RBD residues are labeled grey. A prime symbol indicates residues from the light chain. Dash lines indicate distances within 3.6 Å. (**C**) The β-sheet interaction between Ab08 and RBD. Backbone- and side-chain-mediated H-bonds are indicated by black and orange dash lines, respectively. (**D, E**) Ab08 is compatible with ACE2-binding. (**D**) Structural alignment of ACE2-RBD (PDB ID 6M0J) and Ab08-RBD (this work) reveals no clashes between ACE2 (yellow) and Ab08 (red and blue). (**E**) Ab08 and ACE2 simultaneously bind RBD. A sensor immobilized with RBD was soaked in 100 nM of ACE2 before being further soaked in ACE2-containing buffer with (black) or without (red) 100 nM of Ab08 for BLI signal recording. As a control, the Ab08-RBD interaction was monitored in the absence of ACE2 (blue). (**F**) The distribution of the Omicron mutations. Of the 15 mutations, only one (Q493R) occurs at the epitope but the relative long Cα distance (8.4 Å) between RBD Gln493 and Ab08 Leu55 suggests neglectable impact of Q493R on Ab08-RBD interactions.

To accurately characterize the Ab08 epitope, we crystalized the Ab08 scFv (scAb08) in complex with RBD in the space group of *P2*_1_ and determined the crystal structure to a resolution of 2.8 Å (Fig 3A and S1 Table). The structure was refined to *R*_work_/*R*_free_ of 0.2198/0.2509 without geometry outliers. Interestingly, there existed eight copies of the scAb08: RBD complex related by non-crystallographic symmetry in an asymmetric unit. These copies were overall similar with Cα RMSD ranging from 0.3–0.5 Å. We focused on chain A and chain B for structural description.

We refer to the RBD structure as a high chair with a short backrest. Ab08 bound RBD at one side of the high chair (Fig 3A) away from the receptor-binding motif (RBM) with a buried surface area (BSA) of 1247.9 Å^2^. This epitope overlaps with a few neutralizing antibodies including 35B5, FD20, and S309 that bind to a similar region (S5 Fig). Among the six complementarity-determining regions (CDRs), L-CDR2 made no direct contact with RBD while heavy chain CDR1 (H-CDR1), H-CDR2, H-CDR3, L-CDR1 and L-CDR3 contributed to a BSA of 120.7, 269.9, 404.0, 218.4, and 146.2 Å^2^, respectively. Besides, the framework region was also involved in the binding with a BSA of 88.8 Å^2^. Overall, the interactions involved 18 H-bonds and a number of hydrophobic interactions made by Ile52, Ile54, and Leu55 from H-CDR2, Trp109 from H-CDR3, and Ala352, Ile468, Leu452, Phe 490, and Leu 492 from RBD (Fig 3B). A peculiar feature of Ab08 was that the H-CDR3 aligns with a loop in the ‘backrest’ region, forming an antiparallel β-sheet with 3 backbone-mediated H-bonds (Fig 3C). The β-sheet was further stabilized by 3 H-bonds formed by the side-chains of Ser103, Tyr105 from H-CDR3 and Lys462, Glu465, Arg466 from RBD, and hydrophobic interactions by the side-chains of Trp109 from H-CDR3 and Ile468, Ala352 form RBD (Fig 3C).

Structural alignment of ACE2-RBD and Ab08-RBD showed that ACE2 and Ab08 approached RBD in the opposite directions. Although the Ab08 epitope was adjacent to the RBM, the two did not overlap (Fig 3D). Consistently, the binding between ACE2 and RBD was not affected by Ab08 (Fig 3E).

The Ab08 epitope was relatively conserved. Thus, it covered ~17% of the total RBD residues but only one of the 15 mutations (~7%) from the Omicron strain BA.1 occurred in the Ab08 epitope (Fig 3F). This residue (Gln493) was in the vicinity of Ab08 but its side-chain pointed away from the interface (Fig 3F). Therefore, mutations from the Omicron BA.1 were not expected to affect Ab08 binding, explaining its similar neutralizing efficiency between Omicron BA.1 and Wuhan-Hu-1. Similarly, the additional mutations T376A, D405N and R408S of Omicron BA.2 also did not occur in the Ab08 epitope (S6 Fig) and had no effect in binding (Fig 1D) or neutralization (Fig 1B). Although the three residues that were mutated in the Beta variant were not directly involved in the binding with Ab08, binding affinity between its RBD and Ab08 was reduced by fivefold (Fig 1D). Two mutations, K417N and N501Y are included in the Omicron BA.1, while the E484K mutation is replaced with an alanine mutation in the Omicron strains. This suggests that the bulkier lysine sidechain and/or its positive charge may clash with the nearby *H*-CDR1 of Ab08. Compared with BA.2, BA.4/BA.5 contains a single mutation (L452R) which is also found in the Delta strain. Contrasting to the abovementioned neutral mutations, L452R occurred at the Ab08 epitope, explaining its drastic impact on binding (Fig 1D) and neutralizing activity (S3B Fig).

### Ab08 neutralizes SARS-CoV-2 by destructing the S protein

As Ab08 did not affect the binding of SARS-CoV-2 RBD to the ACE2 receptor, we sought for alternative neutralization mechanisms. Although relatively rare, it has been reported that antibodies can neutralize SARS-CoV-2 by destructing the S trimer [7–9].

**Fig 4:**
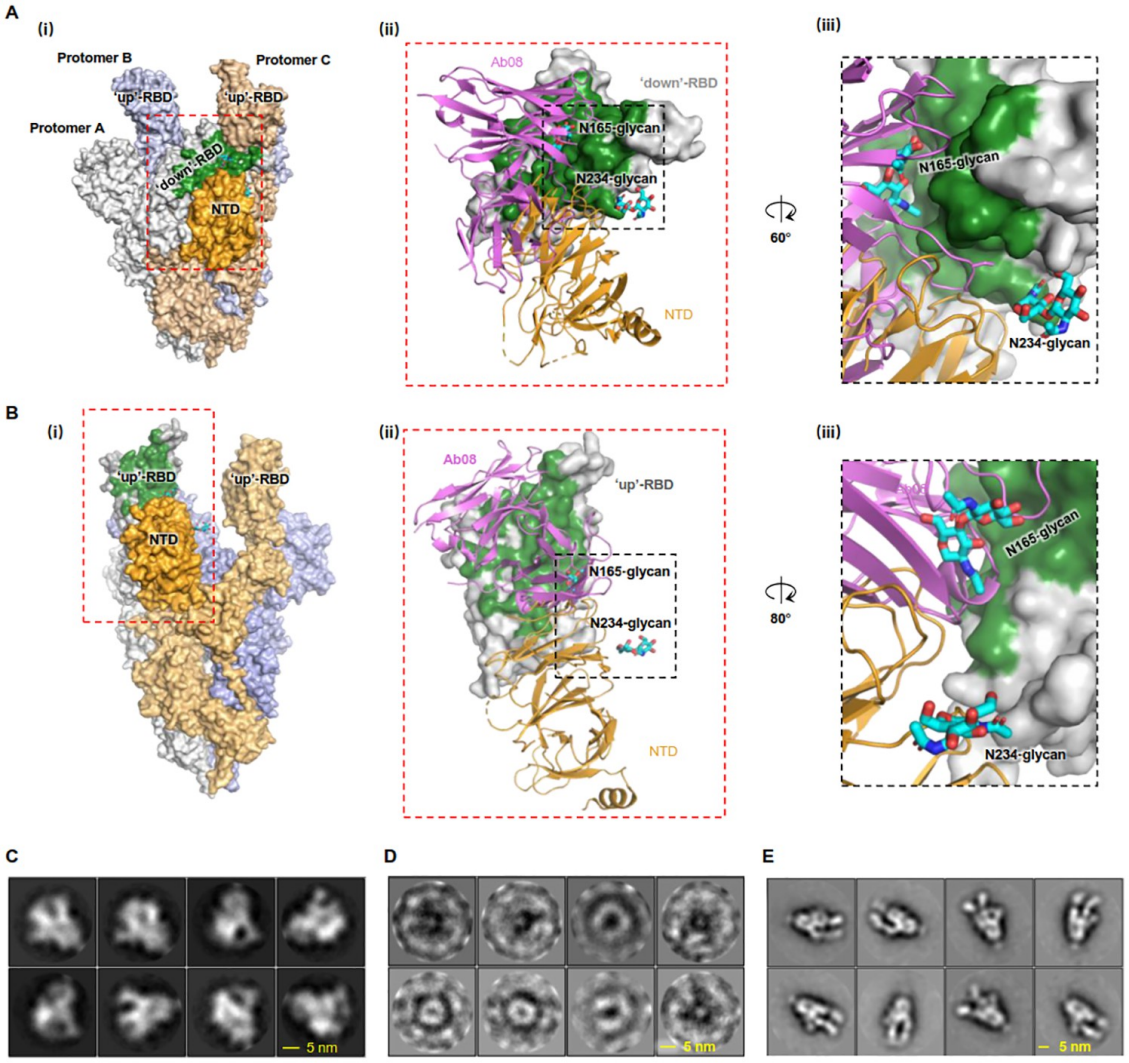
Ab08 neutralizes SARS-CoV-2 by destructing S trimer. (**A-B**) Structural alignment of Ab08 to the S trimer structure (PDB ID 7WLY) reveals severe clashes with the N-terminal domain (NTD) in cases of both the “down”-RBD (A) and the “up”-RBD conformation (B). The epitope of Ab08 is colored green. The three protomers of S and the RBD of the protomer A are color-coded as indicated in (i). In (ii), the RBD from protomer A is shown as surface representation, and Ab08 (magenta) and the NTD from protomer C (orange) are shown as ribbon representations. The two glycans are shown as cyan sticks. In (iii), the disruption of the interactions between NTD N165-linked glycans (Cyan) and RBD by Ab08 is highlighted. (**C-E**) 2D class averages of Negative-staining particles of S-2P showing typical S trimer features (**C**) were lost upon incubation with Ab08 (**D**), but not with an RBM-targeting control mAb (CB6, **E**) [22]. Raw images are in S7 Fig. In **E**, the 2D classes show binding of two CB6 molecules per S trimer and CB6 may induce more “up”-RBD population, a phenomenon that has been reported for other RBM-targeting antibodies [44].

Aligning Ab08 onto the structure of S revealed severe clashes between the antibody and the S protomers in the “open” conformation (Fig 4A), and to a lesser extent in the “close” conformation (Fig 4B). Specifically, Ab08 would compete with the N-terminal domain (NTD) in binding with both the “down”-RBD (in the “close” conformation) and the “up”-RBD (in the “open” conformation). In addition to perturbing the NTD-RBD interactions formed by amino acid residues, Ab08 assumed a position that would compete with the glycan chain on Asn165 for RBD-binding (Fig 4A). Previously, the glycan on Asn165, and that on Asn234 to a lesser extent, have been proposed to play an important role in stabilizing the “open” conformation of S based on binding assays and computational structural analyses [28]. More recently, the glycan chains have been referred as a “glycan lock” that stabilizes the “close” conformation of S in a structural study. The discrepant results may be reconciled with a postulation that the glycans can stabilize both the “open” and the “close” conformation, thus reducing the S conformations in between. In other words, the “close” and the “open” conformations, although not equally favored, represent two energy-minimizing states of S. Because our Ab08 targets to regions where the two glycans bind, it may destabilize both the “open” and “close” conformations of S.

To experimentally investigate whether Ab08 destructs S, the trimer integrity of S was assessed by negative staining. An engineered S named S-2P [29,30] that contains two stabilizing proline mutations was used. As shown in Fig 4C, S-2P particles exhibited characteristic features of a trimer (S7A Fig) with 2D class images resembling the look of “chicken legs”. Such features were gradually lost upon incubation with Ab08 (S7A–S7C Fig), and no recognizable S-2P trimer particles were observed after 1-h treatment (Figs 4D and S7D). As controls, the incubation with buffer (S7E Fig), or with an RBM-targeting antibody (CB6) had no noticeable destruction (Figs 4E and S7F). Therefore, we conclude that Ab08 joins with CR3022 [7], EY6A [8], FD20 [9] and 35B5 [10] as S-destructing neutralizing antibodies.

## Discussion

Antibody-based therapeutics represents important measures to fight COVID-19. However, as the Omicron lineage of SARS-CoV-2 continues to evolve, the emerging Omicron subvariants are not only more transmissible but also more evasive to antibodies. These Omicron subvariants have caused a substantial reduction of neutralizing activity of the sera from vaccinees and escaped the majority of authorized monoclonal antibodies for emergency use [12–16,31]. Thus, Omicron variant-targeting, broadly neutralizing antibodies are urgently needed. In the current study, we have identified Ab08 as one relatively broad-spectrum antibody with therapeutic potential.

Up to now, only a few broadly neutralizing antibodies against Omicron variants have been reported, including LY-CoV1404 (Bebtelovimab) [25], S2K146 [32], and 87G7 [33]. While ACE2-blocking antibodies usually show high neutralizing potency, they are generally more vulnerable to new mutations occurring within the ACE2 binding region. By contrast, the sites outside RBM are subjected to less selective pressures and hence are more conserved. Conforming to this trend, Ab08 identified in the current study targets a relatively conserved epitope outside RBM; consistently, it displays broad activity against several variants of concern/interest including Alpha, Beta, Delta, Gamma, Mu, and Omicron, although the neutralizing activity for Delta and the Omicron BA.4/BA.5 was modest. To cope with the outbreaks by emerging new variants, strategies for enriching antibodies targeting conserved regions should be considered. For example, ACE2-blocking antibodies may be included in counter-selection steps during antibody panning process to enrich binders targeting non-RBM regions. Finally, Ab08 shows therapeutic efficacy in the SARS-CoV-2-infected hACE2 mouse model. The above results indicate that Ab08 alone could have therapeutic potential in clinics. In addition, the structurally characterized epitope will guide the design of non-competing SARS-CoV-2 nAb pairs for potential cocktail therapy.

The mechanistic study has revealed that Ab08 neutralizes SARS-CoV-2 via destructing the S trimer, a mechanism shared by only a few other nAbs [7–10]. Among them, the 35B5 antibody neutralizes the virus by displacing the conserved glycan switch from the RBD and causing the unstable “up” conformation of the RBD and the dissociation of the S trimer [10]. Structural analyses of Ab08 indicate a similar mechanism for Ab08 and reinforces the vulnerability of the glycan-switch site which should be exploited for vaccine design and antibody therapeutic development against SARS-CoV-2.

RBD Leu452 has becoming a hot spot for mutation in recently emerging variants such as BA.4/BA.5 sublineages. As revealed by the crystal structure, Leu452 is at the epitope of Ab08. Leu452 is situated in a hydrophobic microenvironment consisting Leu55 and Ile52 from the heavy chain of Ab08 (S6 Fig). The L452R/Q mutations would introduce unwelcome positive charge/hydrophilic sidechains into this microenvironment and hence weakens binding affinity (Fig 1D). Consistent with this observation, The L452R-containing Delta and Omicron BA.4/BA.5 variant [34] showed modest escape for Ab08 (S3B Fig). To circumvent this, we are currently performing structure-guided antibody optimization of Ab08, with an aim to generate more efficient neutralizing antibodies to combat the SARS-CoV-2 variants with mutations at Leu452 and other sites in the S protein. To this end, we have designed mutations of residues in the vicinity including T33D, K99D, and K99E with an intention to eliminate the electrostatic repulsion and introduce charge-charge interactions.

In summary, we identify a pico-molar nAb against SARS-CoV-2 and variants. The relatively broad activity and *in vivo* efficacy shed promises for the development Ab08 as a potential therapeutic antibody to fight COVID-19.

## Materials and methods

### Ethical statement

This study was approved by the Institutional Review Board of Affiliated Hospital of Nantong University (ID: 2021-K038-01), China. All studies were performed in accordance with the Declaration of Helsinki and written consent was obtained from the Participants.

### RBD^+^ IgG^+^ single B cell sorting and expression of monoclonal antibodies

(Probe conjugation) His-tagged SARS-CoV-2 RBD (Cat: 40592-V08B, Sinobiological) was conjugated with FITC (Cat: ab102884) and (Cat: ab102918) Lightning-Link Conjugation Kits, respectively, according to the manufacture’s procedure (Abcam).

(Isolation) Peripheral B cells were first enriched from blood mononuclear cells (PBMCs) from convalescent patients by removing non-B cells via magnetic depletion (Biotin anti human CD3 [Clone: UCHT1]/CD11b [Clone: M1/70]/CD14 [Clone: HCD14]/CD16 [Clone: 3G8]/CD56 [Clone: HCD56]/CD235a [Clone: HIR2], all from Biolegend and anti Biotin microbeads [Cat: 130-090-485, Miltenyi]) according to the manufacture’s procedure. The enriched B cells were stained with a cocktail of FITC-RBD, PE-RBD, VioBlue anti IgA (Clone: IS11-8E10, Miltenyi), Alexa647 anti IgM (Clone: MHM-88, Biolegend), BV510 anti IgD (Clone: IA6-2, Biolegend), PerCPCy5.5 anti CD3 (Clone: SK7, Biolegend), BV785 anti CD20 (Clone: 2H7, Biolegend) at roome temperature for 10 min. Single CD3^-^CD20^+^IgM^-^IgD^-^IgA^-^FITC-RBD^+^PE-RBD^+^ (corresponding to IgG^+^) B cells were sorted into the 96-well plate containing 4 μL Lysis buffer/well (Lysis buffer: 300 μL Rnasin, 40 U/μL RNase inhibitor, 200 μL 1xDPBS, 400 μL DL-DTT (100 mM), 3100 μL Nuclease-Free Water, all from Thermo) on a FACSMelody cell sorter (BD Biosciences).

(Sequencing) The sequencing of paired heavy chain and light chain was performed as described before [35]. In brief, single RBD^+^IgG^+^ B cell lysate was used as template to perform reverse transcription followed by PCR amplification. PCR products were sequenced and cloned to human IgG1 heavy-chain and IgK/L light-chain vectors to construct mAb heavy or light chain recombinant plasmids respectively.

(Expression) Equal amounts of IgG1 heavy-chain and IgK/L plasmids were transfected into CHO cells by using transfection reagent according to the manufacture’s procedure (Cat. A29133, Thermo Fisher). The culture medium was harvested by centrifugation. The supernatant containing secreted IgG antibodies were filtered, and antibodies were subjected for neutralizing antibody screening. Candidate antibodies were further purified using a Protein A kit (GE Health Science).

### Pseudovirus neutralization assay

(Pseudoviruses production) HEK293T cells were transfected using polyetherimide (PEI) with a plasmid encoding murine leukemia virus (MLV) gag/pol, a retroviral vector encoding EGFP, and an envelope plasmid expressing full-length S protein of SARS-CoV-2 (Wuhan-Hu-1, GenBank: QHD43419.1) and SARS-CoV-2 variants (Plasmids for B.1.1.7 and others were generated by site-directly mutagenesis). Six hours later, the cells were washed and incubated in fresh medium. At 48 hours post-transfection, pseudovirus-containing culture supernatants were harvested.

(Neutralization assay) 100 μL of the pseudoviruses was pre-mixed with 50 μL of serum samples diluted in DMEM and incubated at 37°C for 1 hour (h). The mixture was then onto VeroE6 cells overexpressing hACE2 (denoted as VeroE6-hACE2) pre-seeded in 48-well plates. Eight hours later, the virus/sera-containing media were removed and exchanged with fresh media containing 10% FBS. At 72 hours post-infection, the cells were analyzed by flow cytometry. The infectivity of pseudotyped particles incubated with antibodies was compared with the infectivity observed using pseudotyped particles incubated with DMEM medium containing 2% fetal calf serum (FBS) and standardized to 100%. Average and standard error of mean (SEM, n = 3) were plotted for the experiments which reports data from three independent experiments.

### SARS-CoV-2 authentic virus neutralization assay

A Plaque Reduction Neutralization Test (PRNT) was used to detect SARS-CoV-2 neutralizing Abs. Serially diluted antibodies were mixed with 100 Tissue Culture Infectious Dose (TCID50) of SARS-CoV-2 authentic virus and added to Vero E6 cells to incubate at 37°C in an incubator with 5% CO_2_ for 1 hour. Then a 0.6% agar overlay prepared in cell culture maintenance was applied. At 3 days post infection, a second agar overlay containing 0.2% Neutral Red was applied, and the number of plaques in each sample were recorded after an additional 2 days of incubation. The highest antibody dilution reducing plaque numbers by 50% (PRNT_50_) was calculated.

### Protective efficacy of Ab08 in mice

Specific-pathogen-free, 8 weeks-old male hACE2 mice were obtained from Shanghai Model Organisms Center. The human ACE2 mainly expressed in the lungs, heart, kidneys and intestines of transgenic mice. After being intraperitoneally anaesthetized by 2.5% avertin, the hACE2 mice were inoculated intranasally with SARS-CoV-2 stock virus at a dosage of 10^5^ TCID50, and hACE2 mice intranasally inoculated with an equal volume of PBS were used as a mock-infection control. The infected mice were continuously observed to record body weight and responses to external stimuli and death. Mice were dissected 4 days post infection to collect lungs to screen virus replication and histopathological changes.

### Preparation of homogenate supernatant of lung tissue

Lung homogenates (1 g/mL) were prepared by homogenizing perfused tissues using an electric homogenizer for 2 min 30 seconds (s) in DMEM. The homogenates were centrifuged at 3,000 rpm for 10 min at 4 °C. The supernatant was collected and stored at −80 °C.

### RNA extraction and RT–qPCR for viral loading

Total RNA was extracted from tissues homogenates of organs using the RNeasy Mini Kit (Qiagen), and reverse transcription was performed following the manufacturers’ instructions. qRT-PCR reactions were performed using the PowerUp SYBG Green Master Mix Kit (TAKARA), in which samples were processed in duplicate using the following cycling protocol: 50 °C for 2 min, 95 °C for 2 min, followed by 40 cycles at 95 °C for 15 s and 60 °C for 30 s, and then 95 °C for 15 s, 60 °C for 1 min, 95 °C for 45 s. The primer sequences used for RT–qPCR is targeted against RBS encoding gene of SARS-CoV-2. Standard curve was generated by serial tenfold dilutions of recombinant plasmid with a known copy number (from 109 to 101 copies per μL). Results were expressed as log10-transformed numbers of genome equivalent copies per ml of sample. The cycle threshold (CT) values from samples were plotted on the standard curves, and the number of viral DNA copies per assay was calculated.

### H&E staining

The organs were fixed in 10% buffered formalin solution, and paraffin sections (3–4 μm in thickness) were prepared routinely. The tissues were stained with Harris’ hematoxylin solution for 6 h at a temperature of 60–70 °C and were then rinsed in tap water until the water was colorless. Next, 10% acetic acid and 85% ethanol in water were used to differentiate the tissue 2 times for 2 h and 10 h, and the tissues were rinsed with tap water. In the bluing step, we soaked the tissue in saturated lithium carbonate solution for 12 h and then rinsed it with tap water. Finally, staining was performed with eosin Y ethanol solution for 48 h. Digital images of H&E staining were captured using a Zeiss AxioPlan2 microscope.

### Purification of RBD

RBD was expressed in High Five insect cells and purified using Ni-NTA column as previously described (cite Nat Commun paper please). Briefly, RBD was expressed as a polypeptide with the following sequence from N- to C-: the honey bee melittin signal peptide (KFLVNVALVFMVVYISYIYAA), a Gly-Ser linker, residues of 330–541 of the SARS-CoV-2 S protein (Uniprot P0DTC2), a Gly-Thr linker, the 3C protease site (LEVLFQGP), a Gly-Ser linker, the Avi tag (GLNDIFEAQKIEWHE), a Ser-Gly linker, and a deca-His tag. The protein was secrete expressed and the secretion from 1 L of culture was filtered through a 0.22-μm membrane and incubated with 3.0 mL of Ni-Sepharose Excel beads (Cat 17-3712-03, GE Healthcare) in the presence of 20 mM of imidazole for 2–3 h at 4 °C with mild stirring. The beads were washed with 10 column volume (CV) of 20 mM imidazole in a buffer containing 150 mM NaCl, 20 mM Tris HCl pH 8.0. RBD was eluted using 300 mM of imidazole in the same buffer. After a desalting step (Cat. 732–2010, Bio-Rad), RBD was digested with home-purified His-tagged 3C protease at 1:100 molar ratio (3C protease: RBD) at 4 °C for 16 h. The reaction mix was then passed through a Ni-NTA column which removes 3C protease, undigested RBD, and the cleaved His-tag. The flow-through fractions were collected and concentrated to 8–10 mg/mL.

For biotinylation, the Avi-tagged RBD at 0.8 mg/mL was incubated with 43.5 μM biotin, 10 mM magnesium acetate, 5 mM ATP, and 22 μg/mL home-purified BirA at 4 °C. Biotinylated RBD was concentrated and further fractioned by gel permeation.

### Purification of Ab08 Fab fragment

Ab08 IgG1 was expressed as secretion in Expi293F cells (Cat. A14527, Thermo Fisher). Plasmids carrying DNA encoding the heavy chain and the light chain (all with a signal peptide for secretion) were used for transient expression. One liter of Expi293F cells at a density of 2 × 10^6^ per milliliter were transfected with 1.33 mg of the plasmid carrying the light chain, 0.67 mg plasmid carrying the heavy chain, and 4 mg polyethylenimine. VPA was added to a final concentration of 2 mM to aid expression. The medium containing secreted Ab08 IgG1 was collected 48–60 hours post-transfection, centrifuged at 3,000 × g and filtered through a 0.22-μm membrane, before being incubated with Protein A beads (Smart-Lifesciences) for 2 h at 4°C. The beads were washed with 20 CV of buffer A (20mM Tris-HCl pH7.5, 150 mM NaCl) and eluted with 0.1 M glycine pH 3.0, 150 mM NaCl. The eluent was immediately neutralized by 1 M Tris-HCl pH 8.0. Purified Ab08 IgG1 was buffer-exchanged into Buffer A using a desalting column (Bio-Rad).

To generate Fab for the negative staining assay, Ab08 IgG1 purified above was digested using immobilized papain (Sigma) to release Fab. The immobilized papain was activated with Digest Buffer (20 mM cysteine, 2mM EDTA, 150 mM NaCl, 20 mM Tris-HCl pH 7.5) at 37°C for 10 min. The papain was added to IgG in digesting buffer at a molar ratio of 1:100 (papain:IgG) and incubated at 37 °C for 4 h. The mix was buffer-exchanged to Buffer A on a desalting column. The Fc fragment was removed by binding with Protein A beads and the Ab08 Fab was collected in the flow-through fraction.

### Production of Ab08 (scFv) fragment

Single chain variable fragments (scFvs) were expressed in Escherichia coli MC1061 cells with a pelB signal peptide (SKYLLPTAAAGLLLLAAQPAMA) at the N-terminus, the variable domain of heavy chain, a GS linker (15 amino acids), the variable domain of heavy chain and a Myc and hexahistidine tag at the C terminus. Cells carrying the plasmid were grown in Terrific Broth (TB, 0.017 M KH_2_PO_4_, 0.072 M K_2_HPO_4_, 1.2% (w/v) tryptone, 2.4% (w/v) yeast extract and 0.4% (v/v) glycerol) at 37°C to OD_600_ of 0.5 at 37°C. The growth temperature was then lowered to 22°C for 1.5 h and the cells were induced with 0.02% (w/v) arabinose for 16 h. Cells from 1 L of culture were resuspended in 20 mL of TES buffer (0.5 M sucrose, 0.5 mM EDTA, and 0.2 M Tris-HCl pH 8.0). After 0.5 h of dehydration, cells were rehydrated abruptly with 40 mL of ice-cold MilliQ H_2_O at 4 °C for 1 h. The periplasmic extract was collected by centrifugation at 20,000 × g at 4 °C for 30 min. The crude extract was adjusted to contain 150 mM of NaCl, 2 mM of MgCl_2_, and 20 mM of imidazole before being incubated with 1 mL of Ni-NTA resin. After batch binding for 2 h at 4°C, the beads were washed with 30 mM of imidazole in Buffer A and eluted with 300 mM imidazole in buffer A. For crystallization, the protein was further purified by gel filtration.

### Expression and purification of SARS-CoV-2 S protein

The plasmid for mammalian transient expression of S protein was kindly provided by Prof. Yao Cong at the authors’ institute. The pcDNA 3.1+ plasmid harbors the mammalian codon-optimized gene encoding residues Met1-Gln1208 of the SARS-CoV-2 S with mutations K986P, V987P, a GSAS linker substituting Arg682-Arg685 (the furin cleavage site), a C-terminal T4 fibritin trimerization motif (GYIPEAPRDGQAYVRKDGEWVLLSTFL), a TEV protease cleavage site, a FLAG tag, and a polyhistidine tag [36]. For expression, 0.2 L of Expi293F cells at a density of 2 × 10^6^ per milliliter were transfected with a mixture containing 0.2 mg of plasmid and 0.4 mg of polyethylenimine and cultured for 2.5 days at 37°C. The supernatant was collected by centrifugation at 3,000 × g for 15 min, cleared by filtration through a 0.22-μm membrane, and adjusted to contain 200 mM NaCl, 20 mM imidazole, 4 mM MgCl_2_, and 20 mM Tris-HCl pH 7.5. The mixture was added with 1 mL of Ni-NTA beads and incubated at 4 °C for 2 h for batch binding. The beads were poured into a gravity column and washed with 20 mM of imidazole in Buffer B (200 mM NaCl and 20 mM Tris-HCl pH 7.5) and eluted with 250 mM imidazole in Buffer B. The eluted fractions were pooled and imidazole was removed using a desalting column (Bio-Rad). Protein was concentrated using a 100-kDa cutoff membrane concentrator.

### Crystallization

For crystallization, RBD which was purified by the ourselves was mixed with 1.2-fold molar concentration of Ab08 (scFv) and the mixture was loaded onto a Superdex Increase 200 10/300 GL column for gel permeation. Fractions containing the complex were pooled, concentrated to 20 mg/mL for crystallization. The Ab08-RBD complex (150 nL) was mixed with 150 nL of precipitant solution in a sitting drop plate with 60 μL of reservoir using a Gryphon LCP robot. The plates were incubated at 20 °C for crystal growth in a RockImager 1000 (Formulatrix). The crystallization condition contained 0.06 M MgCl_2_, 0.06 M CaCl_2_, 12.5% (v/v) MPD, 12.5% PEG (w/v) 1,000, 12.5% (w/v) PEG 3,350, 0.1 M MOPS, and 0.1 M HEPES pH 7.5. Crystals were harvested at day 15 using a MiTeGen loop and cryo-cooled in liquid nitrogen before X-ray diffraction data collection.

### X-ray diffraction data collection and structure determination

X-ray diffraction data were collected on a Pilatus 6 M detector at beamline BL18U1 at the Shanghai Synchrotron Radiation Facility using a 50 × 50 μm X-ray beam with a wavelength of 0.9791 Å. Data were integrated with XDS [37] and scaled using Aimless [38]. The structure was solved by molecular replacement using Phaser [39] with the appropriate part of PDB entry 7CYV as search models [9]. The initial model was built using Phenix.autobuild [40]. The model was adjusted manually in Coot [41] using 2Fo-Fc maps and refined using Phenix. Structure was visualized using PyMOL (Molecular Graphics System, Version 2.0 Schrödinger, LLC).

### Bio-layer interferometry assay

The binding kinetics were measured using a bio-layer interferometry (BLI) assay on an Octet RED96 machine (ForteBio). Biotinylated prototype or different variant RBDs (2 μg/mL) were immobilized on a streptavidin sensor (Cat. 18–5019) in the presence of Buffer C (0.05% (v/v) Tween 20 in PBS buffer) at 30°C. The sensor was equilibrated (baseline) for 120 s, before being incubated with Ab08 (scFv) at various concentrations (association) for 360 s. The sensor was then moved into buffer C for dissociation and the signal was monitored for another 600 s. Data were fitted for a 1:1 stoichiometry for *K*_D_, *k*_on_, and *k*_off_ calculations using the built-in software Data Analysis 10.0. Only the binding kinetics for the scFv version was reported.

To test cross-competition between Ab08 (scFv) and ACE2, 2 μg/mL of Biotinylated RBD was immobilized on a streptavidin sensor. After saturated using 100 nM of ACE2, the sensor was incubated with either the mixture of 100 nM Ab08 and ACE2 or 100 nM of ACE2 alone.

RBD for the Wuhan-Hu-1 strain was purified in house as described above. Biotinylated RBD for other variants were purchased from Sino Biological (Cat. 40592-V08H85-B; Cat. 40150-V08B2-B) and antibodies-online GmbH (Cat. ABIN7193998). The procedures were all the same for different RBD. Because the only RBD mutation of the Delta variant, L452R, is included in BA.4/BA.5, we did not perform BLI assay for the Delta variant. Also, we note that BA.4 and BA.5 share the same RBD sequence.

### Negative staining electron microscopy and 2D classification

S-2P trimer at 30 μg/mL was either incubated with Buffer B or with Fab (Ab08 or CB6) at a molar ratio of 1:6 (S trimer: Fab) at 4 °C. The samples were applied to a copper grid with a supporting carbon film (Cat BZ31024a, Beijing Zhongjingkeyi Technology) which had been treated by H2 and O2 for 20 s in a plasma cleaner (Model 950 Advanced Plasma System, Gatan). The grid was then stained with 5% (w/v) uranyl acetate. The samples were imaged using a Tecnai G2 Spirit electron microscopy (Thermo Fisher) operated at 120 kV. Electron microscopic data were collected on a 4K × 4K CCD Eagle camera, at a nominal magnification of 67,000, which corresponds to a physical pixel size of 1.74 Å.

Negative staining particles were automatically picked using EMAN 2.2 [42]. Particles were imported to Relion 3.1 [43] for 2D classification. A total number of 11,463 particles from 50 micrographs were used for the 2D classification of the S alone; 13,960 particles from 50 micrographs were used for the 2D classification of FD20-treated S; 17,152 particles from 70 micrographs were used for the 2D classification of CB6-treated S.

### Statistical analysis

Statistical analysis was performed using GraphPad Prism 8.0 (GraphPad Software, United States). Specific statistical methods were described in figure legend. Comparison between two groups were performed using unpaired Student’s *t*-test. Data was reported as mean ± SEM, and error bars indicate SEM. P < 0.05 was regarded as statistically significant.

## Supporting information

S1 Table Data collection and refinement statistics. (DOCX)

S1 Fig Identification of Ab08 from a convalescent donor. Antibodies were harvested from the medium of recombinant CHO cells and used for neutralizing assays against pseudoviruses (Wuhan-Hu-1).
(TIF)

S2 Fig Neutralization effect of CB6 against the Omicron BA.1 pseudovirus. (TIF)

S3 Fig Neutralization assay of Ab08 using SARS-CoV-2 pseudoviruses. (**A**) S protein schematic with mutations found in B.1.1.7 (Alpha), B.1.351 (Beta), P.1 (Gamma), B.1.617.2 (Delta), B.1.621 (Mu), and indicated Omicron variants relative to ancestral SARS-CoV-2. (**B**) Lentiviruses pseudotyped with SARS-CoV-2 S proteins from Wuhan-Hu-1, Alpha, Beta, Delta, Gamma, Mu and Omicron BA.4/BA.5 were incubated with serial dilutions of Ab08, and IC_50_ was determined. IC_50_ values are indicated as μg/mL in brackets.
(TIF)

S4 Fig Neutralization assay of Ab08 with various point mutations of SARS-CoV-2 D614G pseudoviruses. Lentiviruses pseudotyped with various key point mutations of SARS-CoV-2 D614G S including L452R, L452Q and T478K were incubated with serial dilutions of Ab08, and IC_50_ was determined. IC_50_ values are indicated as μg/mL in brackets.
(TIF)

S5 Fig Comparison of Ab08 epitope with those of existing antibodies that bind to a similar region. **(A**) The epitope of Ab08 (green) on RBD. (**B-D**) The overlap (red) of the epitope of Ab08 (green) with the epitopes from the indicated antibodies (cyan).
(TIF)

S6 Fig Structural insights into the different activity of Ab08 against SARS-CoV-2 variants. The distribution of RBD mutations (magenta sphere) from the Beta (**A**), Omicron BA.2 (**B**), and Omicron BA.4/BA.5 (**C**) variants in the context of the Ab08 epitope. RBD (grey) and Ab08 (pink for the heavy chain and blue for the light chain) are shown as ribbon representations. The expanded view in C highlights the interaction between Leu452 of RBD and indicated Ab08 residues. The structural illustration is not provided for the Delta strain because the only RBD mutation of this variant is L452R which is included in BA.4/BA.5.
(TIF)

S7 Fig Incubation with Ab08 destructs S-2P trimer. (**A-D**) Negative-staining images of S-2P upon incubation with Ab08 (Fab) at indicated time points. (**E, F**) Typical negative staining image of S-2P treated with PBS buffer (**E**) or CB6 (**F**) for 60 min. Red circles highlight typical S-2P particles.
(TIF)
