# Supplementary material for: A Spike-destructing human antibody effectively neutralizes Omicron-included SARS-CoV-2 variants with therapeutic efficacy
Source: PLoS Pathog. 2023 Jan 27;19(1):e1011085. doi: 10.1371/journal.ppat.1011085 (PMC9907810; doi:10.1371/journal.ppat.1011085)
Supplement: S1 Table — (DOCX) [file ppat.1011085.s001.docx]

**Supporting information**

**S1 Table.** Data collection and refinement statistics.

|  | Ab08-RBD |
| --- | --- |
| **Data collection** |  |
| Space group | *P 1 2_1_ 1* |
| Cell dimensions |  |
| *a*, *b*, *c* (Å) | 92.64, 116.74, 180.72 |
| *α,β, γ* (°) | 90.00, 90.31, 90.00 |
| Wavelength (Å) | 0.97915 |
| Resolution (Å) | 49.39 – 2.80 (2.85 - 2.80)*^a^* |
| *R*_merge_ | 0.161 (0.945) |
| *R*_pim_ | 0.071 (0.433) |
| *I*/σ*I* | 9.4 (1.9) |
| Completeness (%) | 99.3 (98.7) |
| Multiplicity | 6.9 (6.5) |
| *CC** *^b^* | 0.993 (0.928) |
|  |  |
| **Refinement** |  |
| Resolution (Å) | 44.93 – 2.80 |
| No. reflections | 9,381 |
| *R*_work_ / *R*_free_ | 0.2198/0.2509 |
| No. atoms | 26,586 |
| Protein | 26,369 |
| Ligands | 196 |
| Water | 21 |
| No. residues | 3,474 |
| B-factors (Å^2^) | 56.40 |
| Protein | 56.10 |
| Ligand/ion | 97.85 |
| Water | 47.03 |
| R.m.s deviations |  |
| Bond lengths (Å) | 0.003 |
| Bond angles (°) | 0.55 |
| Ramachandran |  |
| Favoured (%) | 97.75 |
| Allowed (%) | 2.25 |
| Outlier (%) | 0 |
| **PDB ID** | 7WQV |

*^a^*Highest resolution shell is shown in parenthesis. *^b^CC**$=\sqrt{\frac{{2CC}_{1/2}}{1+{CC}_{1/2}}}$
