# Supplementary figures and images for: A Spike-destructing human antibody effectively neutralizes Omicron-included SARS-CoV-2 variants with therapeutic efficacy
Source: PLoS Pathog. 2023 Jan 27;19(1):e1011085. doi: 10.1371/journal.ppat.1011085 (PMC9907810; doi:10.1371/journal.ppat.1011085)

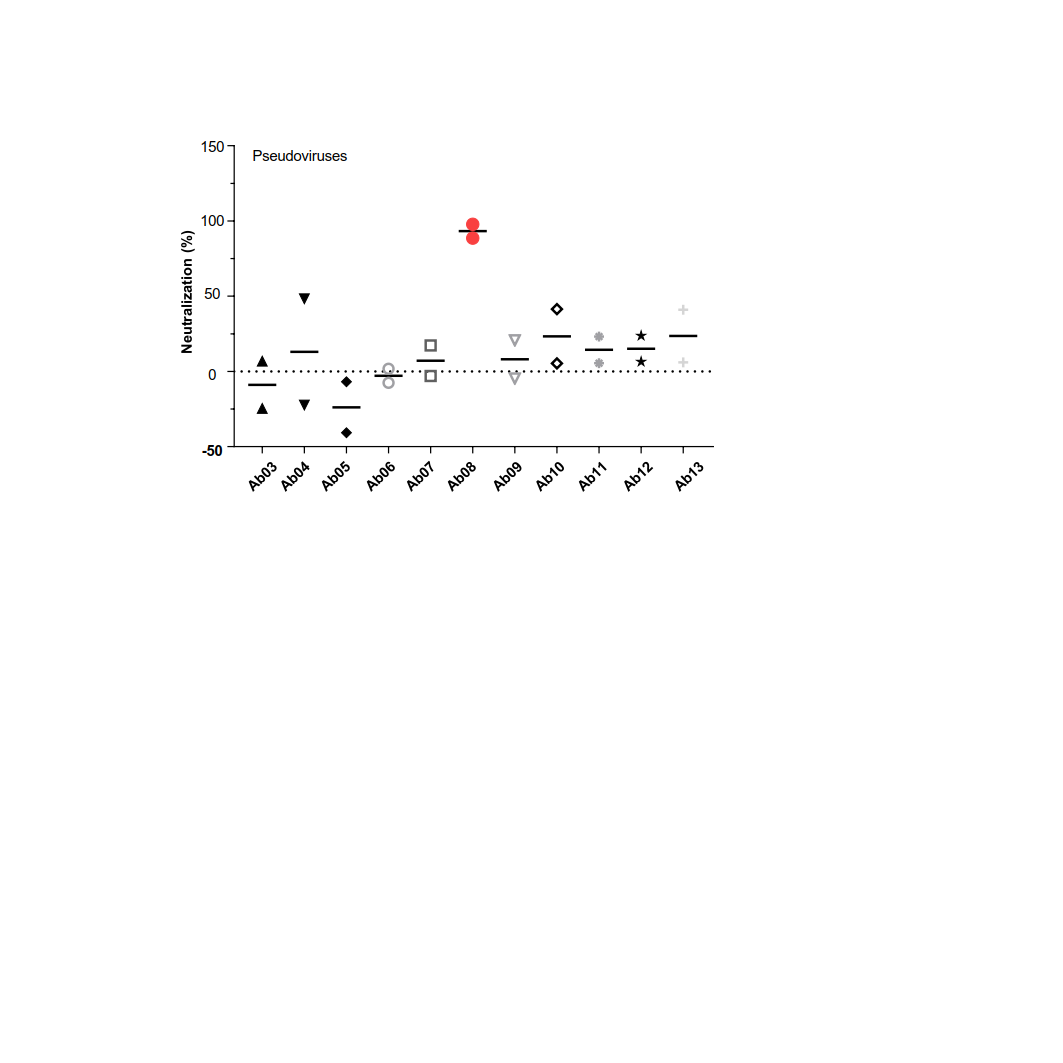

Supplement: S1 Fig — Antibodies were harvested from the medium of recombinant CHO cells and used for neutralizing assays against pseudoviruses (Wuhan-Hu-1). (TIF) [file ppat.1011085.s002.tif]

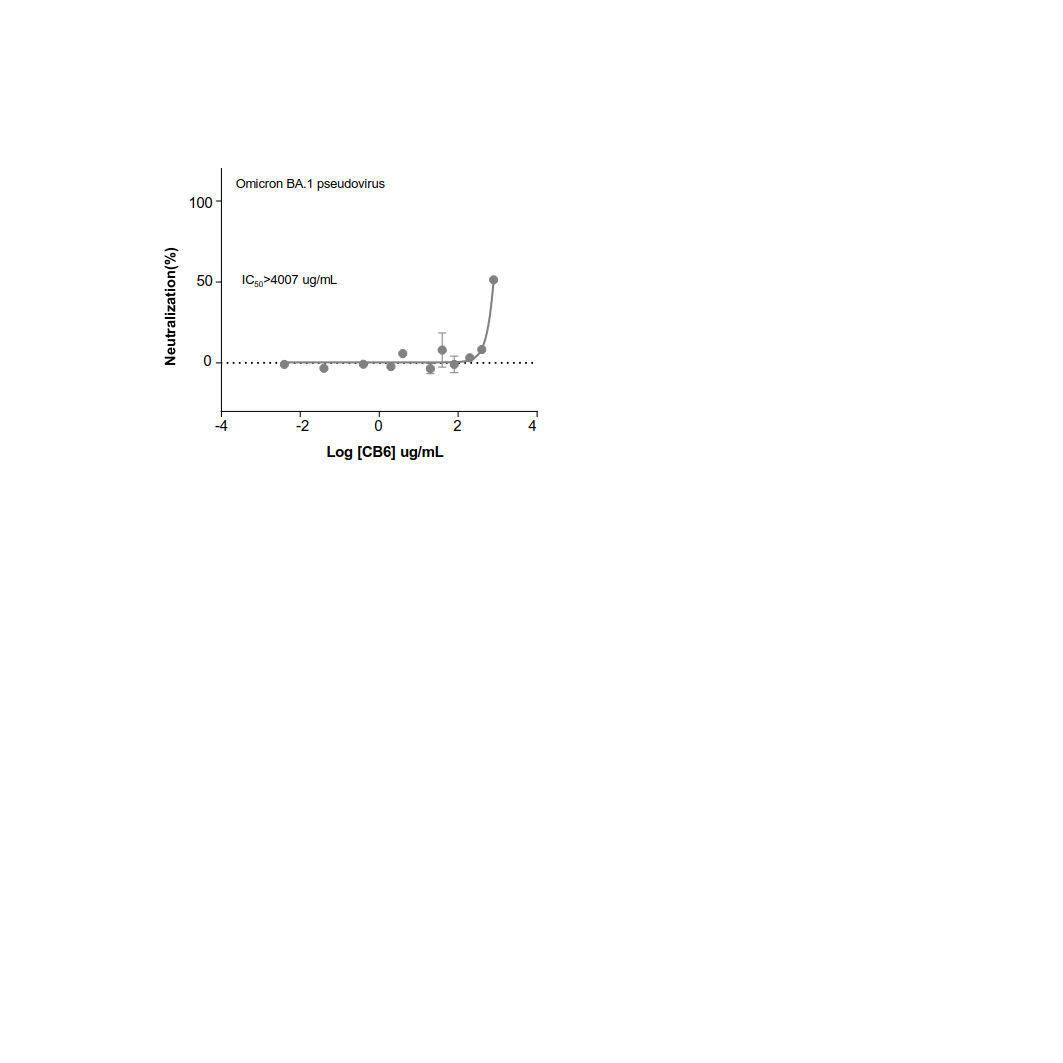

Supplement: S2 Fig — (TIF) [file ppat.1011085.s003.tif]

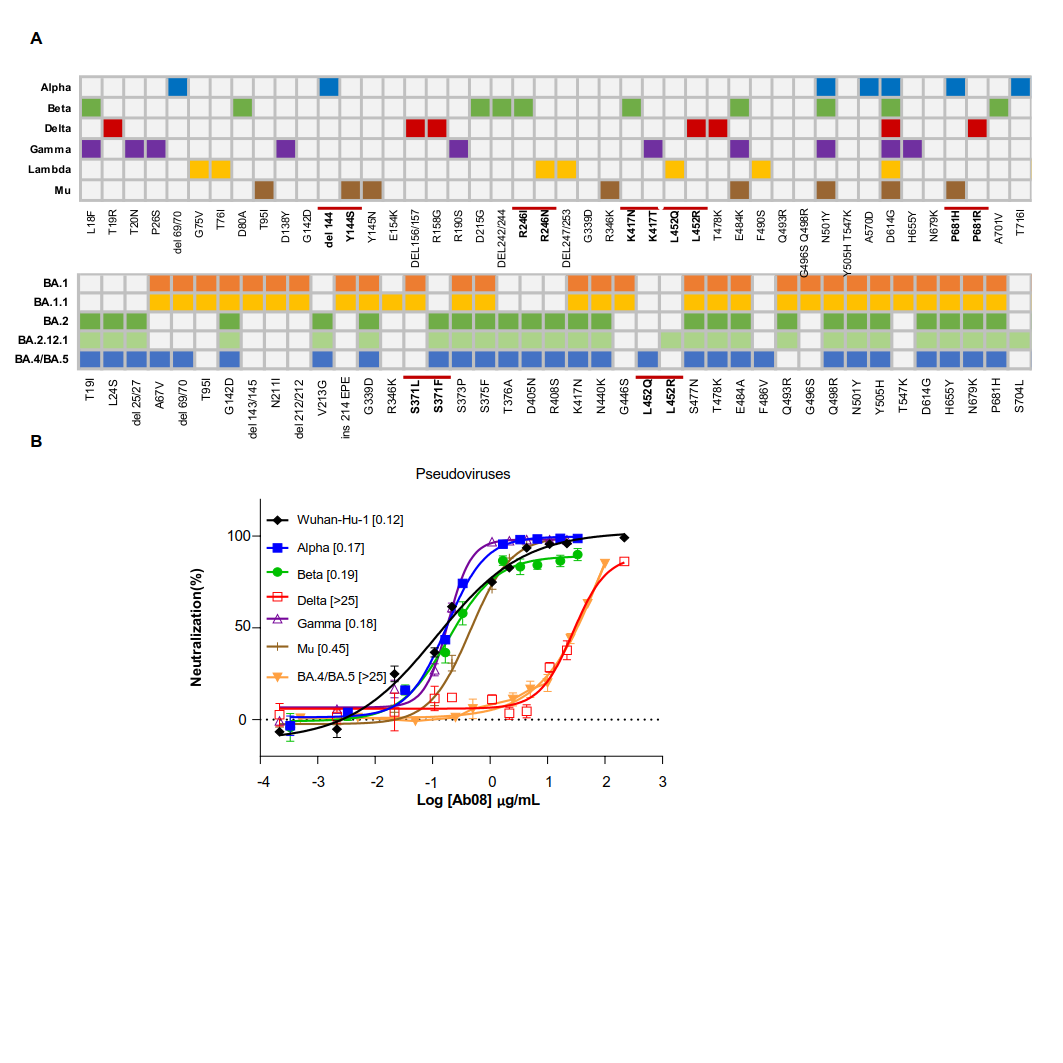

Supplement: S3 Fig — (A) S protein schematic with mutations found in B.1.1.7 (Alpha), B.1.351 (Beta), P.1 (Gamma), B.1.617.2 (Delta), B.1.621 (Mu), and indicated Omicron variants relative to ancestral SARS-CoV-2. (B) Lentiviruses pseudotyped with SARS-CoV-2 S proteins from Wuhan-Hu-1, Alpha, Beta, Delta, Gamma, Mu and Omicron BA.4/BA.5 were incubated with serial dilutions of Ab08, and IC50 was determined. IC50 values are indicated as μg/mL in brackets. (TIF) [file ppat.1011085.s004.tif]

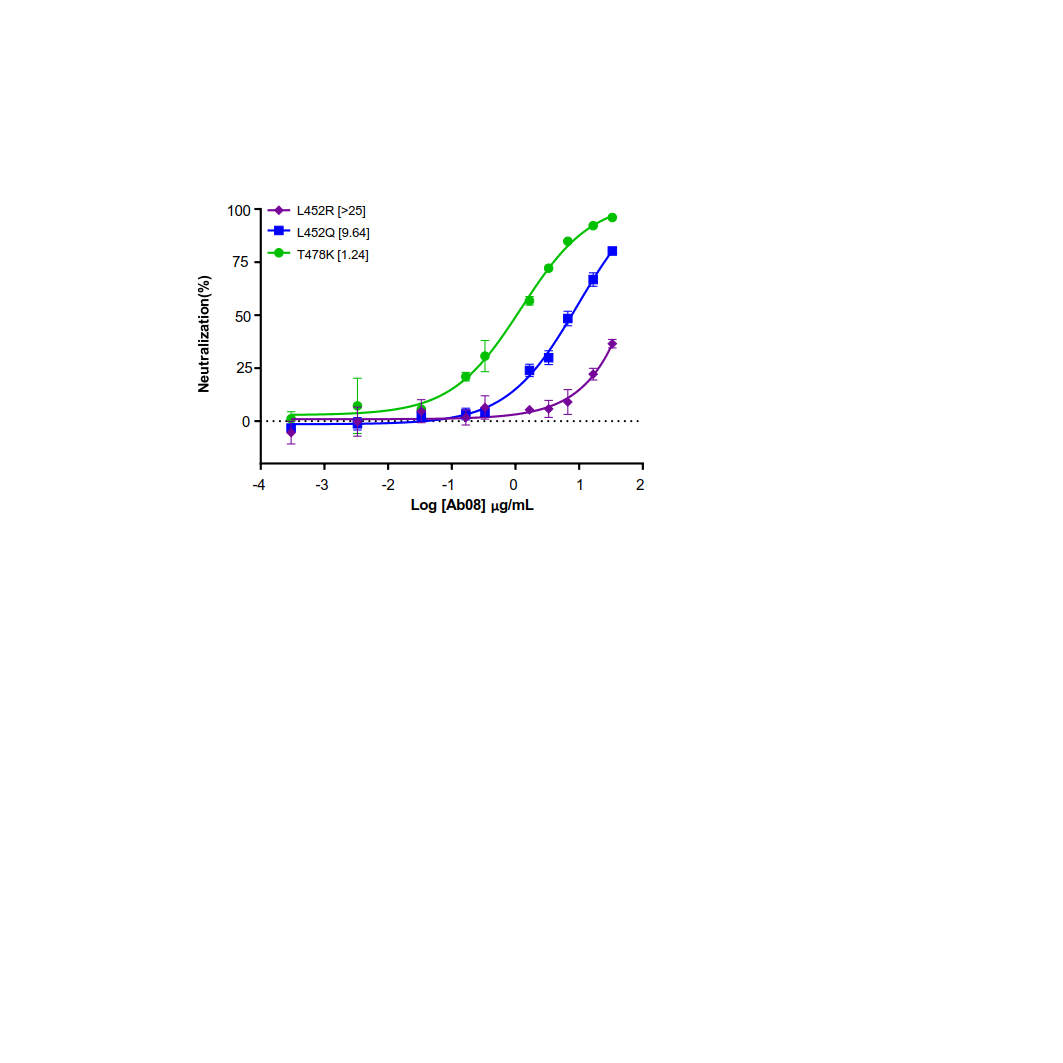

Supplement: S4 Fig — Lentiviruses pseudotyped with various key point mutations of SARS-CoV-2 D614G S including L452R, L452Q and T478K were incubated with serial dilutions of Ab08, and IC50 was determined. IC50 values are indicated as μg/mL in brackets. (TIF) [file ppat.1011085.s005.tif]

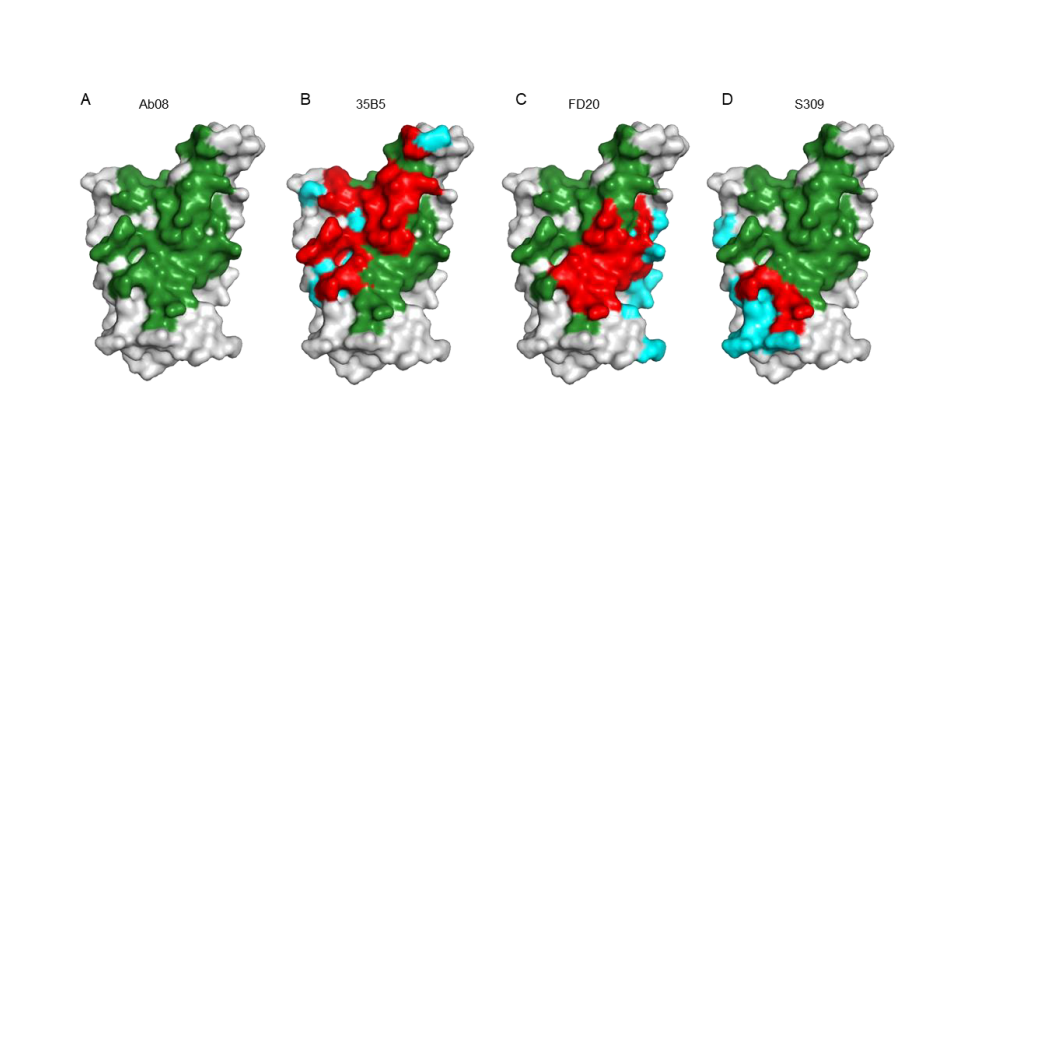

Supplement: S5 Fig — (A) The epitope of Ab08 (green) on RBD. (B-D) The overlap (red) of the epitope of Ab08 (green) with the epitopes from the indicated antibodies (cyan). (TIF) [file ppat.1011085.s006.tif]

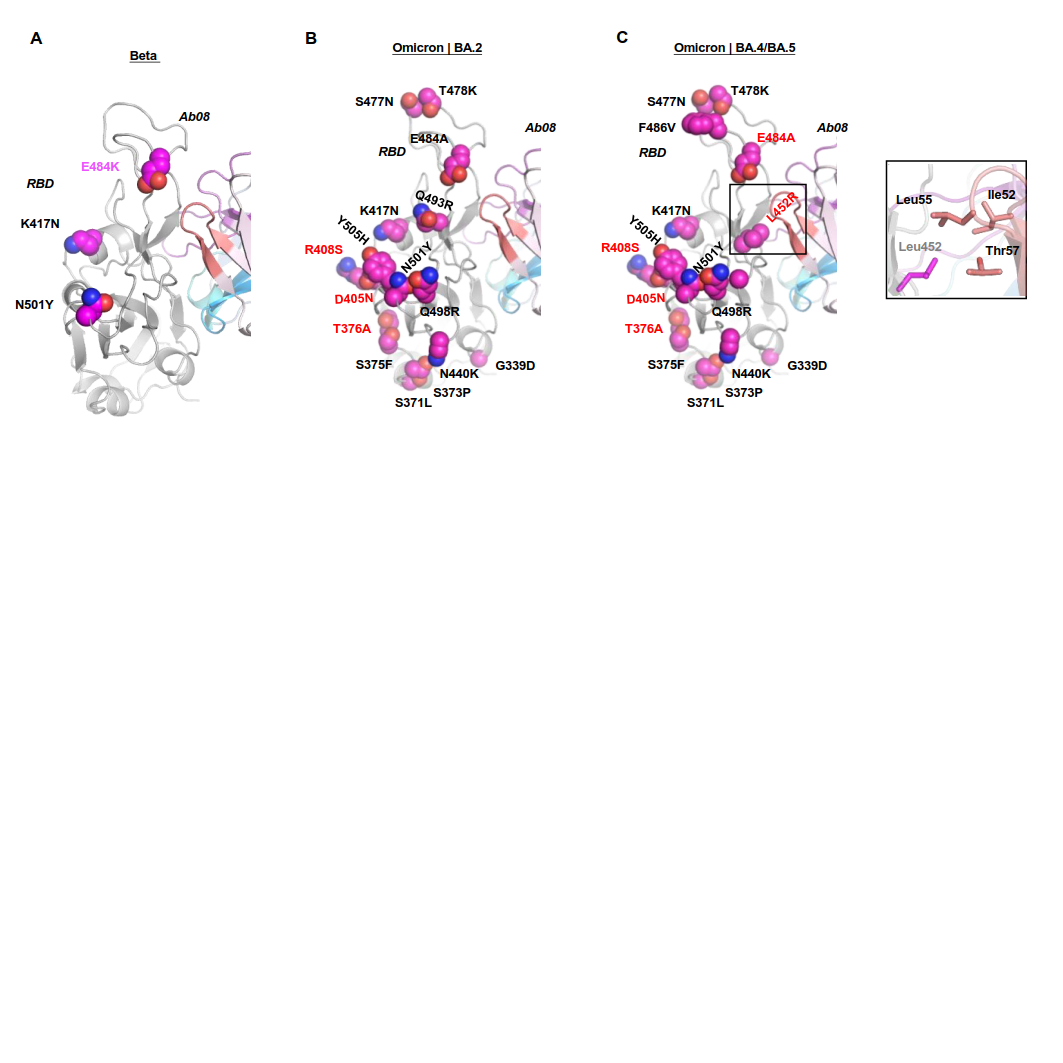

Supplement: S6 Fig — The distribution of RBD mutations (magenta sphere) from the Beta (A), Omicron BA.2 (B), and Omicron BA.4/BA.5 (C) variants in the context of the Ab08 epitope. RBD (grey) and Ab08 (pink for the heavy chain and blue for the light chain) are shown as ribbon representations. The expanded view in C highlights the interaction between Leu452 of RBD and indicated Ab08 residues. The structural illustration is not provided for the Delta strain because the only RBD mutation of this variant is L452R which is included in BA.4/BA.5. (TIF) [file ppat.1011085.s007.tif]

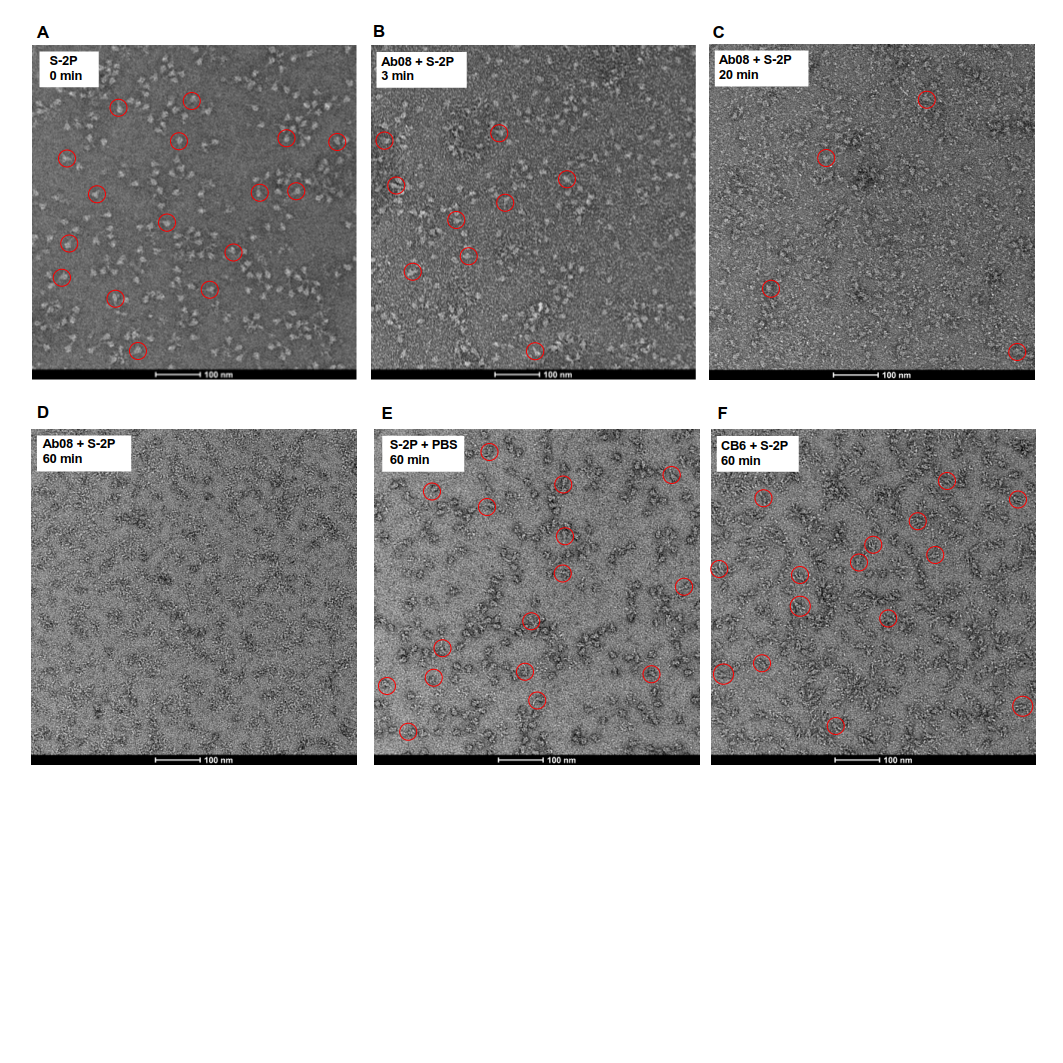

Supplement: S7 Fig — (A-D) Negative-staining images of S-2P upon incubation with Ab08 (Fab) at indicated time points. (E, F) Typical negative staining image of S-2P treated with PBS buffer (E) or CB6 (F) for 60 min. Red circles highlight typical S-2P particles. (TIF) [file ppat.1011085.s008.tif]
